# Molecular Mechanism of the Cell Death Induced by the Histone Deacetylase Pan Inhibitor LBH589 (Panobinostat) in Wilms Tumor Cells

**DOI:** 10.1371/journal.pone.0126566

**Published:** 2015-07-15

**Authors:** Tao Yan-Fang, Li Zhi-Heng, Xu Li-Xiao, Fang Fang, Lu Jun, Li Gang, Cao Lan, Wang Na-Na, Du Xiao-Juan, Sun Li-Chao, Zhao Wen-Li, Xiao Pei-Fang, Zhao He, Su Guang-Hao, Li Yan-Hong, Li Yi-Ping, Xu Yun-Yun, Zhou Hui-Ting, Wu Yi, Jin Mei-Fang, Liu Lin, Ni Jian, Hu Shao-Yan, Zhu Xue-Ming, Feng Xing, Wang Jian, Pan Jian

**Affiliations:** 1 Department of Hematology and Oncology, Children's Hospital of Soochow University, Suzhou, China; 2 Department of Gastroenterology, the 5th Hospital of Chinese PLA, Yin chuan, China; 3 Department of Cell and Molecular Biology, Cancer Institute (Hospital), Chinese Academy of Medical Sciences, Peking Union Medical College, Beijing, China; 4 Translational Research Center, Second Hospital, The Second Clinical School, Nanjing Medical University, Nanjing, China; Institute of Biochemistry and Biotechnology, TAIWAN

## Abstract

**Background:**

Wilms tumor (WT) is an embryonic kidney cancer, for which histone acetylation might be a therapeutic target. LBH589, a novel targeted agent, suppresses histone deacetylases in many tumors. This study investigated the antitumor activity of LBH589 in SK-NEP-1 and G401 cells.

**Methods:**

SK-NEP-1 and G401 cell growth was assessed by CCK-8 and in nude mice experiments. Annexin V/propidium iodide staining followed by flow cytometry detected apoptosis in cell culture. Gene expressions of LBH589-treated tumor cells were analyzed using an Arraystar Human LncRNA Array. The Multi Experiment View cluster software analyzed the expression data. Differentially expressed genes from the cluster analyses were imported into the Ingenuity Pathway Analysis tool.

**Results:**

LBH589 inhibited cell proliferation of SK-NEP-1 and G401 cells in a dose-dependent manner. Annexin V, TUNEL and Hochest 33342 staining analysis showed that LBH589-treated cells showed more apoptotic features compared with the control. LBH589 treatment inhibited the growth of SK-NEP-1 xenograft tumors in nude mice. Arraystar Human LncRNA Array analysis of genes and lncRNAs regulated by LBH589 identified 6653 mRNAs and 8135 lncRNAs in LBH589-treated SK-NEP-1 cells. The most enriched gene ontology terms were those involved in nucleosome assembly. KEGG pathway analysis identified cell cycle proteins, including *CCNA2*, *CCNB2*, *CCND1*, *CCND2*, *CDK4*, *CDKN1B* and *HDAC2*, etc. Ingenuity Pathway Analysis identified important upstream molecules: *HIST2H3C*, *HIST1H4A*, *HIST1A*, *HIST1C*, *HIST1D*, histone H1, histone H3, *RPRM*, *HSP70* and *MYC*.

**Conclusions:**

LBH589 treatment caused apoptosis and inhibition of cell proliferation of SK-NEP-1and G401 cells. LBH589 had a significant effect and few side effects on SK-NEP-1 xenograft tumors. Expression profiling, and GO, KEGG and IPA analyses identified new targets and a new “network” of genes responding to LBH589 treatment in SK-NEP-1 cells. *RPRM*, *HSP70* and *MYC* may be important regulators during LBH589 treatment. Our results provide new clues to the proapoptotic mechanism of LBH589.

## Introduction

Wilms tumor (WT) is an embryonic cancer of the kidney composed of blastemal, stromal and epithelial elements. WT is also the most common malignant neoplasm of the urinary tract in children [1]. The overall 5-year survival is estimated as > 80% [4]; however, for individuals, the prognosis is highly dependent on individual staging and treatment. Although WT is almost curable, with long-term survival, the combination of chemotherapy, radiotherapy and surgery often results in severe complications in adulthood [2]. Therefore, decreases the treatment burden and improve outcome of patients are still required [3]. We evaluated the efficacy of LBH589, a histone deacetylases (HDACs) pan inhibitor to inhibit WT development *in vitro* and *in vivo*.

HDACs are a large family of enzymes involved in chromatin remodeling that have crucial roles in numerous biological processes, largely through repressing transcription [4–6]. For its importance in regulating gene expression, histone acetylation is now being investigated as therapeutic targets [7–9]. Different HDAC inhibitors induce cell death of cancer cells with different mechanisms, including alterations of both histone and non-histone proteins and changes in gene expression [10, 11]. 2–10% of important biological processes genes were changed when inhibition of HDACs [12]. In addition to modifying histones, HDACs also target many other non-histone protein substrates to regulate gene expression [13, 14]. Recently, HDACs have received increasing attention because HDAC-inhibiting compounds are being developed as promising anti cancer therapeutics.

Several structurally diverse inhibitors for HDACs which have been developed as anti cancer therapeutic agents *in vitro* and *in vitro*, they cause differentiation, cell cycle arrest or apoptosis [15–19]. Maintenance growth of cell and differentiation is very highly dependent on tight and coordinated transcriptional regulation of genes. Recently, research has led to the development of HDAC inhibitors as novel anticancer agents. In addition to their effect on epigenetic mechanisms, HDAC inhibitors also can change the acetylation state of many cellular proteins involved in oncogenic processes, resulting in antitumor effects [20–23].

HDAC inhibitor LBH589 has demonstrated antitumor activity, including HDACs suppression and induction of tumor cell apoptosis in various human cancer models. In Triple-negative breast cancer (TNBC), LBH589 has anti-proliferative properties in aggressive breast cancer refractory to hormonal therapy [24]. In human renal cell carcinoma, LBH589 caused obvious cell cycle arrest in the G2/M phase and caused cell apoptosis [25]. In prostate cancer, the combined treatment of LBH589 and radiation therapy induced more apoptosis and led to a steady increase of sub-G_1_ population and abolishment of radiation therapy-induced G_2_/M arrest [26]. In the deadliest skin cancer, melanoma, low nanomolar concentration of LBH589 inhibited the growth of all melanoma cell lines [27]. Treatment of high-risk neuroblastoma cell lines with LBH589 resulted in dose-dependent growth arrest and apoptosis[28]. In hepatocellular carcinoma, LBH589 significantly inhibited HCC growth and metastasis *in vitro* and *in vivo* [29]. In oral squamous cell carcinoma, LBH589 induces apoptosis through regulation of specificity protein 1 (Sp1) in oral squamous cell carcinoma cell lines. LBH589 significantly reduced cell growth and the sub-G_1_ cell population and induced apoptosis [30]. In cisplatin- resistance ovarian cancer, a combination of cisplatin and LBH589 could overcome cisplatin-associated resistance in ovarian cancer cells, in the presence of low-dose LBH589 [31]. In small-cell lung cancer (SCLC), multicenter, nonrandomized, phase 2 trials were designed to evaluate the antitumor activity of LBH589 in patients with previously treated SCLC. Modest clinical activity of LBH589 combined with a favorable safety profile in pretreated SCLC patients was observed [32].

Until now, there has been no report of an antitumor effect of LBH589 in WT. The aim of this study was to analyze the antitumor effect and molecular function of LBH589 in human WT cells and in xenograft models.

## Materials and Methods

### Cell and culture conditions

SK-NEP-1 and G401 Human kidney (Wilm's Tumor) cell line obtained from the American Type Culture Collection (ATCC) was maintained in the Maccyo’5 (Life Technologies Inc., Gaithersburg, MD, USA) supplemented with 20% heat-inactivated fetal bovine serum (Invitrogen Co., NY, USA) in a humidified incubator with 5% CO_2_ at 37°C. LBH589 (Cat: S1030 Selleck Chemicals, West Paterson, NJ, USA) was dissolved in DMSO (Cat: D4540 Sigma–Aldrich, St. Louis, MO, USA)

### Cell proliferation

Cell proliferation analysis was introduced before [3]. SK-NEP-1 and G401 cells (2 × 10^4^) were seeded in 96-well plates overnight and incubated with DMSO, 1 nM LBH589, or increasing concentrations of LBH589 (0.01–10.0 μM) for 24 hours. The same volume of DMSO was added to the vehicle treated wells. Each drug concentration was performed at least in four replicate wells. Then, 10 μL CCK8 (Cell Counting Kit-8: CK04-13, Dojindo Molecular Technologies, Inc. Minato-ku, Tokyo; JAPAN) solution was added to each well, incubated at 37°C for 4 h and the optical density (OD) values were measured at 450 nm using a scanning multi-well spectrophotometer (Bio Rad Model 550, Hercules, California; USA). Compared with control group, relative survival rate was calculated from the absorbance values. Cell proliferation was calculated as a percentage of the DMSO- treated control wells with 50% inhibitory concentration (IC_50_) values derived after plotting proliferation values on a logarithmic curve. The IC_50_ of LBH589 inhibitor was calculated by Graph Prism software.

### Cell cycle analysis

Cell cycle analysis was introduced before [3]. Briefly, cells were collected and washed for 5 minutes with PBS by centrifugation at 125 × g. Then, cells were fixed with paraformaldehyde and permeabilized with 0.5% Triton X-100. Next, cells were resuspended in staining solution, 1.5 μmol/L propidium iodide (P4170, Sigma–Aldrich, St. Louis, MO, USA) and 25 μg/ml RNase A and incubated at 37°C for half a hour. The samples (1 ×10^4^ cells) were analyzed by flow cytometry with a Beckman Gallios Flow Cytometer.

### Apoptosis assay

Apoptosis analysis was introduced before [3]. Cellular apoptosis assay was according to the manufactory of BD Annexin V Staining Kit (Cat: 556420, BD Biosciences, Franklin Lakes, NJ USA). Briefly, cells were washed twice with cold PBS and then resuspend in 1× binding buffer at concentration of about 1 × 10^6^ cells/ml. Then 100 μl of the solution (~1 ×10^5^ cells) was transferred to a 5 ml culture tube. Annexin V and PI 5 μl/test was added. Cells were gently mixed and incubated at RT in the dark for 15 min. Then 400 μl of 1× binding buffer was added to each tube. Cells were analyzed by flow cytometry within 1 hour.

### Western blot analysis

Western blot analysis was introduced before [3].Cellular proteins were extracted in 40 mM Tris–HCl (pH 7.4) containing 150 mM NaCl and 1% (v/v) Triton X-100, supplemented with protease inhibitors. Equal amounts of protein were resolved on 12% SDS-PAGE gels, and then transferred to a PVDF membrane (Millipore, Bedford, MA). Blots were blocked and then probed with antibodies against Caspase 3 (1:1000, Cell Signaling Technology, Inc. Danvers, MA), PARP (1:1000, Cell Signaling Technology, Inc. Danvers, MA), RPRM(1:1000, GTX110976, GeneTex, Inc. Irvine, CA, USA), c-Myc (1:1000, Cell Signaling Technology, Inc. Danvers, MA), PRKCA (1:1000, BOSTER, Wuhan, China), DNAJA3(1:1000, GeneTex, Inc. Irvine, CA, USA), acetyl-Histone H3 (Lys9) (1:1000, Cell Signaling Technology, Inc. Danvers, MA), acetyl-Histone H4 (Lys8) (1:1000, Cell Signaling Technology, Inc. Danvers, MA), BCL2 (1:1000, Abcam Trading (Shanghai) Company Ltd. Pudong, Shanghai, China.), Caspase 9 (1:1000, Cell Signaling Technology, Inc. Danvers, MA), Histone H3(1:1000, Santa Cruz Biotechnology, Inc. Dallas, Texas, USA) and GAPDH (1:5000, Sigma, St. Louis, MO). After three times’ washing, blots were then incubated with horseradish peroxidase (HRP) conjugated secondary antibodies and visualized by enhanced chemiluminescence kit (Pierce, Rockford, IL). Protein bands were visualized after exposure of the membrane to Kodak X-ray film.

### Hoechst 33342 staining analysis

Cells were seeded into 6-well plates and then treated with LBH589 (50nM or 100nM) and cultured at 37°C for 24 hours, stained with 0.1 μg/ml Hoechst 33342 (Sigma, St. Louis, MO, USA) for 5 min, then observed with filters for blue fluorescence under fluorescence microscopy (OLYMPUS IX71; Olympus Corporation, Tokyo, Japan). Abnormal nuclear cells were counted between the LBH589 treatment group and DMSO control group.

### Analysis of apoptosis by TUNEL assay

TUNEL is a common method which can detect DNA fragmentation. DNA double-strand breaks happened late in the apoptotic cells and can be assessed using the TUNEL Apoptosis Detection Kit (Cat: KGA704; Kengent, Nanjing, China). TUNEL analysis was introduced before[33]. Apoptotic cells were photographed by fluorescence microscopy (OLYMPUS IX71; Olympus Corporation, Tokyo, Japan).

### Xenograft assays the treatment effect of LBH589 in nude mice

We carried out this study strictly accordant with the recommendations in the Guide for the Care and Use of Laboratory Animals of the National Institutes of Health. The protocol has been approved by the Committee on the Ethics of Animal Experiments of Soochow university (Permit Number: 2013-05-03). The mouse study was ended when tumor growth to sizes up to 4000mm^3^. 1 × 10^7^ SK-NEP-1 cells were subcutaneously injected into five nude mice every group. 10 days after injection, mice were treatment with PBS, DMSO, and LBH589 10mg/kg and 20mg/kg dose three times per week. And the treatment last six weeks. During the six weeks these mice were examined for subcutaneous tumor growth and health condition three times per week. The tumor volumes were calculated according to this formula: volume = length × width^2^/2. After the last treatment, the mice were killed under sodium pentobarbital anesthesia and the tumor weight was measured.

### Analyze the genes and LncRNAs related with LBH589 treatment with LncRNA array (Arraystar Human LncRNA ArrayV3.0)

SK-NEP-1 cells were treated with 100nM LBH589 and control group cells were treated with the same volume of DMSO 24 hours later. Human LncRNA Array analysis was performed by KangChen Bio-tech, Shanghai P.R. China. And experimental details were introduced by Yu et al [34]. Briefly, RNA purified from total RNA after removal of rRNA was amplified and transcribed into fluorescent cRNA and cDNA was labeled and hybridized to the Human LncRNA Array v3.0 (8660 K, Arraystar). 30,586 LncRNAs and 26,109 coding transcripts which collected from the most authoritative databases such as RefSeq, UCSC, Knowngenes, Ensembl and many related literatures can be detected by the microarray.

### Gene ontology analysis and KEGG pathway analysis

Gene ontology (GO) analysis functionally analyze the differentially expressed genes with GO categories which were derived from Gene Ontology (http://david.abcc.ncifcrf.gov/summary.jsp). Pathway analysis of the differentially expressed genes was performed based on the latest Kyoto Encyclopedia of Genes and Genomes (KEGG) database (http://www.genome.jp/kegg). We performed ontologic pathway enrichment analysis for the differently expressed genes and gene product enrichment with particular attention to GO biological processes and molecular function (*P*-value ≤ 0.05).

### Ingenuity pathway analysis (IPA)

Our datasets representing genes with altered expression profile derived from array analyses were imported into the Ingenuity Pathway Analysis Tool (IPA Tool; Ingenuity H Systems, Redwood City, CA, USA; http://www.ingenuity.com).

IPA analysis was introduced before [3]. IPA Tool allows the identification of biological networks, global functions and functional pathways of a particular dataset.

### Real-time PCR analysis certification of dyes-regulated genes in LBH589-treated SK-NEP-1 cells

Quantitative real-time PCR was performed to determine the expression levels of dyes-regulated genes in LBH589-treated SK-NEP-1 cells. Real-time PCR analysis was introduced before [3]. cDNA synthesis was performed on 4 ug of RNA in a 10 ul sample volume using SuperScript II reverse transcriptase (Invitrogen Co., NY, USA) as recommended by the manufacturer. Reactions were run on Light cycler 480 using the universal thermal cycling parameters. The real time PCR primers used to quantify *GAPDH* expression were: F: 5′-AGAAGGCTGGGGCTCATTTG-3′ and R: 5′-AGGGGCCATCCACAGTCTTC-3′; *RPRM* were: F: 5′- GCATGAGGACTTTCAGAGGG-3′ and R: 5′- GCAAACCTGTCGGAGTCAAT -3′; *DHRS2* were: F: 5′- CTCCATGTAGGGCAGCAACT-3′ and R: 5′- GTAGGGAGCACTCTGGGGAC-3′; *DNAJA3* were: F: 5′-TGTGGAAGGAGGCAGTACAA -3′ and R: 5′- TGCGTCTTCCCTGACCTCT -3′; *STMN2* were: F: 5′- CGGGTAAAAGCAAGAGCAGA-3′ and R: 5′- TCTGCACATCCCTACAATGG -3′; *PRKACA* were: F: 5′-ATCCAAGTGGGCTGTGTTCT-3′ and R: 5′- GAGTGATGGCTTCCAACTCC -3′; *PAM* were: F: 5′- GAAGGCTTCCTCATCCACTG -3′ and R: 5′- TTTTGCATTGGATATTCGCA -3′; *PTPN7* were: F: 5′-TCGGATGTAGTTGGCATTGA-3′ and R: 5′- CCTCCAAGGACCGATACAAG -3′ for *EIF2AK2* were: F: 5′- ACTTGGCCAAATCCACCTG-3′ and R: 5′- CCCAGATTTGACCTTCCTGA -3. Expression of genes was normalized to endogenous *GAPDH* expression.

### Statistical analysis

Each experimental condition was performed two or three times, and these replicates were presented in results. All values are presented as means ± SEM. Student’s paired t-test was applied to reveal statistical significances. P values less than 0.05 were considered significant. Statistical analyses were performed using SPSS Software for Windows (version 11.5; SPSS, Inc., Chicago, IL).

## Results and Discussion

### Growth inhibitory effect of LBH589 on SK-NEP-1 and G401 cells

**Fig 1:**
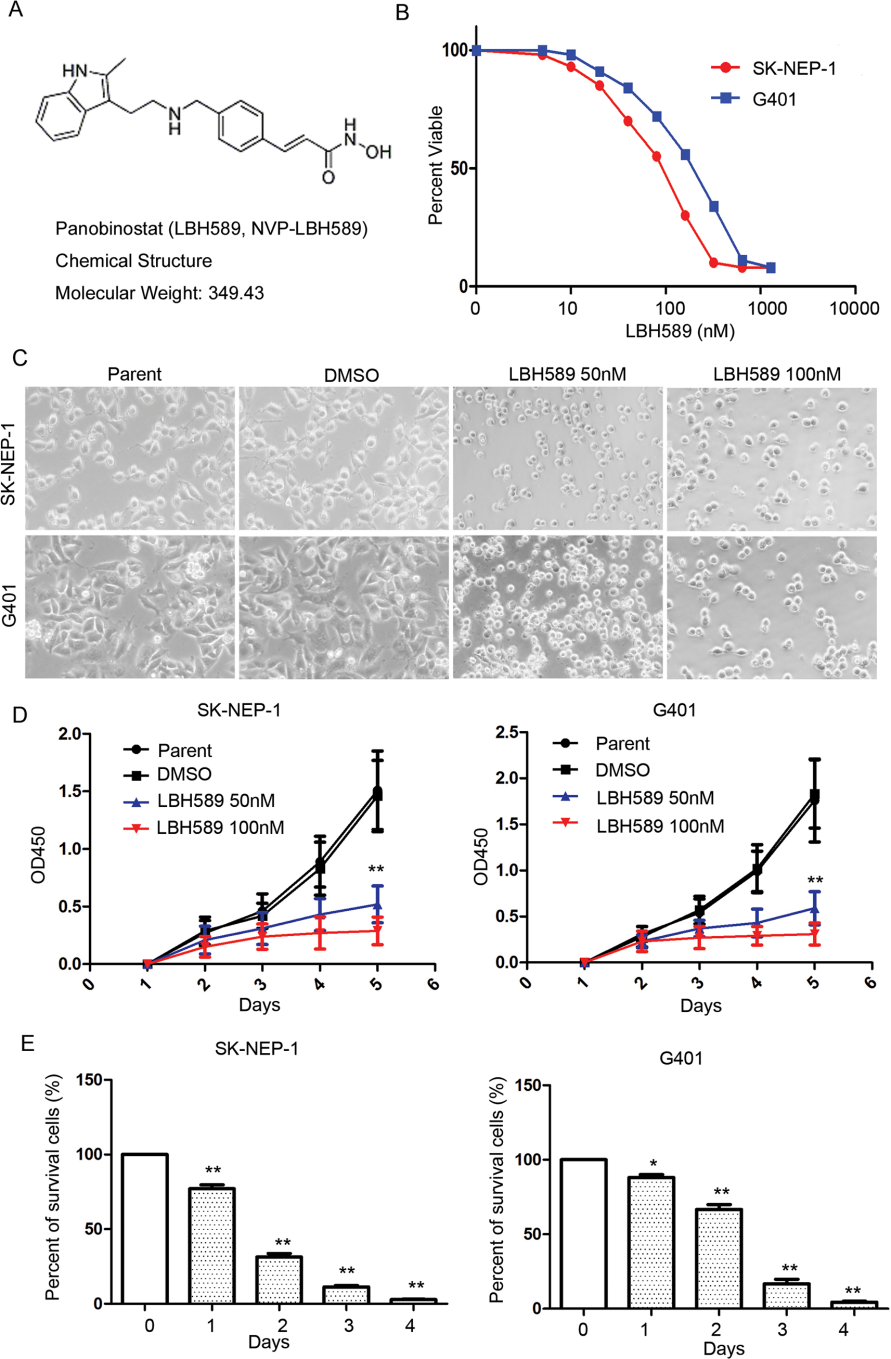
Growth inhibitory effect of LBH589 on SK-NEP-1 and G401 cells. (A) Molecular Structure of LBH589. (B) Proliferation and IC_50_ analysis of LBH589. Experiments were performed in quadruplicate and repeated two times. IC_50_s: SK-NEP-1, 74.15 nM; and G401, 147.3 nM. (C) Micrographs were taken of SK-NEP-1 and G401 cells treated with LBH589 (50nM and 100 nM) or DMSO. (D) Proliferation analysis of SK-NEP-1 and G401 cells treated with 50nM and 100nM LBH589. A CCK-8 assay showed that the inhibition rate at 5 days post-treatment was SK-NEP-1 cells: 81.4 ± 8.21% and G401: 83.1 ± 6.6% compared with the DMSO control group. (E) Cell survival analysis of SK-NEP-1 and G401 cells treated with 50nM LBH589 for 1–4 days. *<0.05,** P < 0.01.

LBH589 treatment resulted in inhibition of cell proliferation of SK-NEP-1 and G401 cells in a dose-dependent manner (Fig 1A and 1B). The IC_50_ of LBH589 for SK-NEP-1 cells was 76.34 nM and for G401 was 143.02 nM. The morphology of SK-NEP-1 and G401 cells changed significantly under LBH589-treatment for 48 hours at concentrations of 50nM and 100nM (Fig 1C). Proliferation analysis showed that LBH589 treatment significantly inhibited cell proliferation (Fig 1D). A CCK-8 assay showed that the inhibition rate at 5 days post-treatment was 81.4 ± 8.21% in SK-NEP-1 cells and 83.1 ± 6.6% G401 cells compared with the DMSO control group (*P* < 0.01). Fig 1E showed that cell survival was significantly reduced when SK-NEP-1 and G401 cells treated with 50nM LBH589 for 1–4 days.

### LBH589 induced apoptosis in SK-NEP-1 and G401 cells

**Fig 2:**
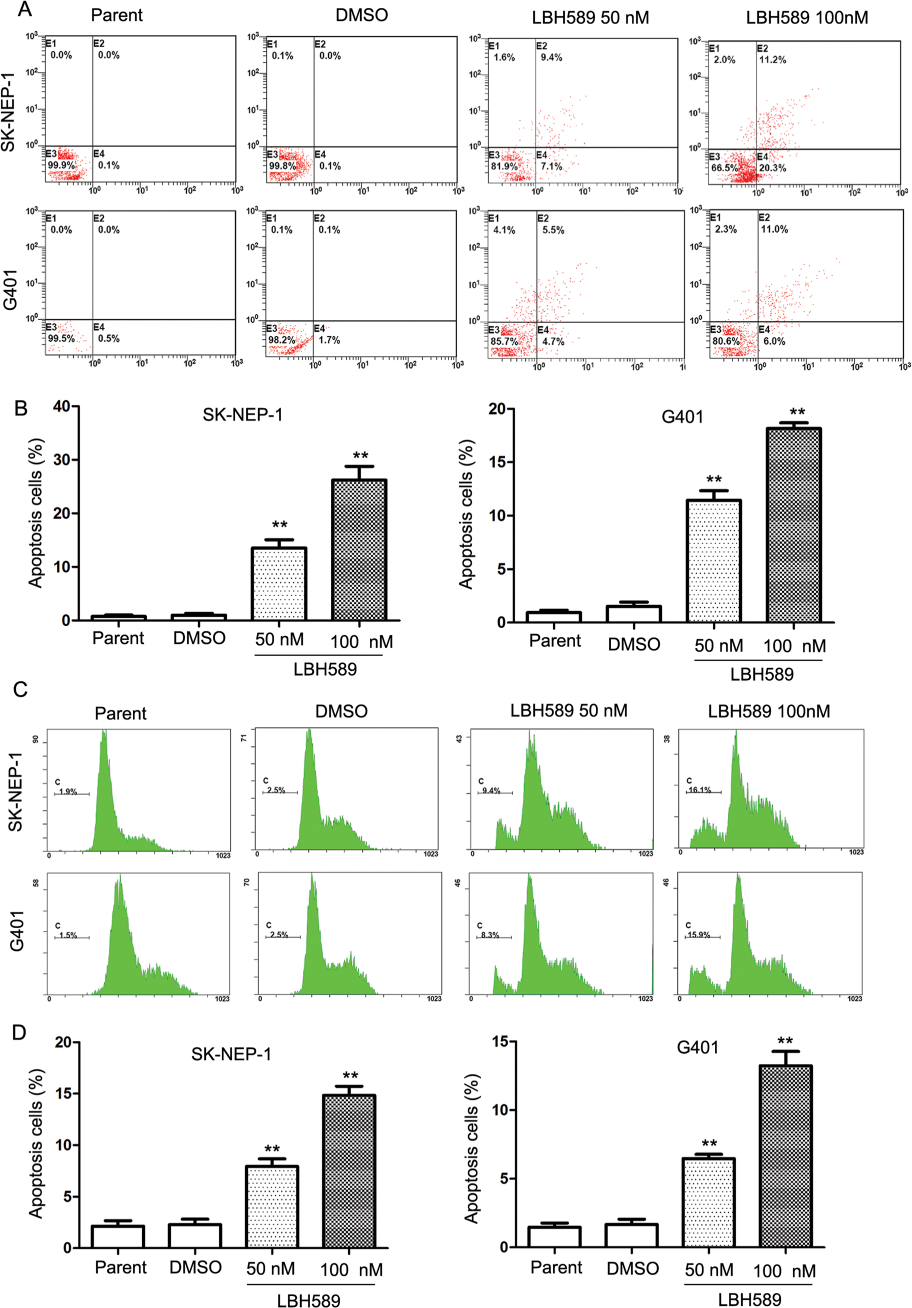
Apoptosis analysis of SK-NEP-1 and G401 cells induced by LBH589. (A) Annexin V staining of cells following 24 h treatment with LBH589 at 50nM or 100nM compared with DMSO control mock treatment. In the LBH589 treatment group, many more cells showed apoptotic features compared with the control group. (B) The proportion of apoptotic cells in the LBH589-treated cells was significantly greater than that in the DMSO control group: SK-NEP-1 (100nM 26.2% ± 4.45% *vs*. DMSO 0.97% ± 0.62%, respectively; *P* = 0.009); and G401 (100nM 18.17% ± 0.90% *vs*. DMSO1.53 ± 0.67%, respectively; *P* < 0.001). (C) Cell late apoptosis analysis of SK-NEP-1 and G401 were treated with 50 nM and 100 nM LBH589. As expected, DNA fragmentation was observed after LBH589 treatment and increased in a dose dependent manner. (D) Cell late apoptosis showed significant apoptosis in SK-NEP-1 (100nM 14.83% ± 1.55% *vs*. DMSO 2.30% ± 0.92%, respectively; *P* = 0.001); and G401 cells (100nM 13.23% ± 1.79% *vs*. DMSO1.67 ± 0.67%, respectively; *P* = 0.003).These analyses were repeated three times. * *P* < 0.05; ** *P* < 0.01.

To confirm whether LBH589 induces apoptosis in SK-NEP-1 and G401 cells, we used Annexin V, cell cycle, tunnel and Hochest33342 assays, and activation of PARP in SK-NEP-1 and G401 cells after LBH589 treatment. The result showed that among cells treated with LBH589 50nM and 100nM for 24 hours, many more cells showed apoptotic feature compared with control group, for both SK-NEP-1 and G401 cells (Fig 2A and 2B). The proportion of apoptotic cells in the LBH589-treated cells was significantly greater than that in the DMSO control group: SK-NEP-1 (100nM 26.2% ± 4.45% *vs*. DMSO 0.97% ± 0.62%, respectively; *P* = 0.009); and G401 (100nM 18.17% ± 0.90% *vs*. DMSO1.53 ± 0.67%, respectively; *P* < 0.001). Cell late apoptosis was also analyzed (Fig 2C and 2D), and the results showed significant apoptosis in SK-NEP-1 (100nM 14.83% ± 1.55% *vs*. DMSO 2.30% ± 0.92%, respectively; *P* = 0.001); and G401 cells (100nM 13.23% ± 1.79% *vs*. DMSO1.67 ± 0.67%, respectively; *P* = 0.003).

**Fig 3:**
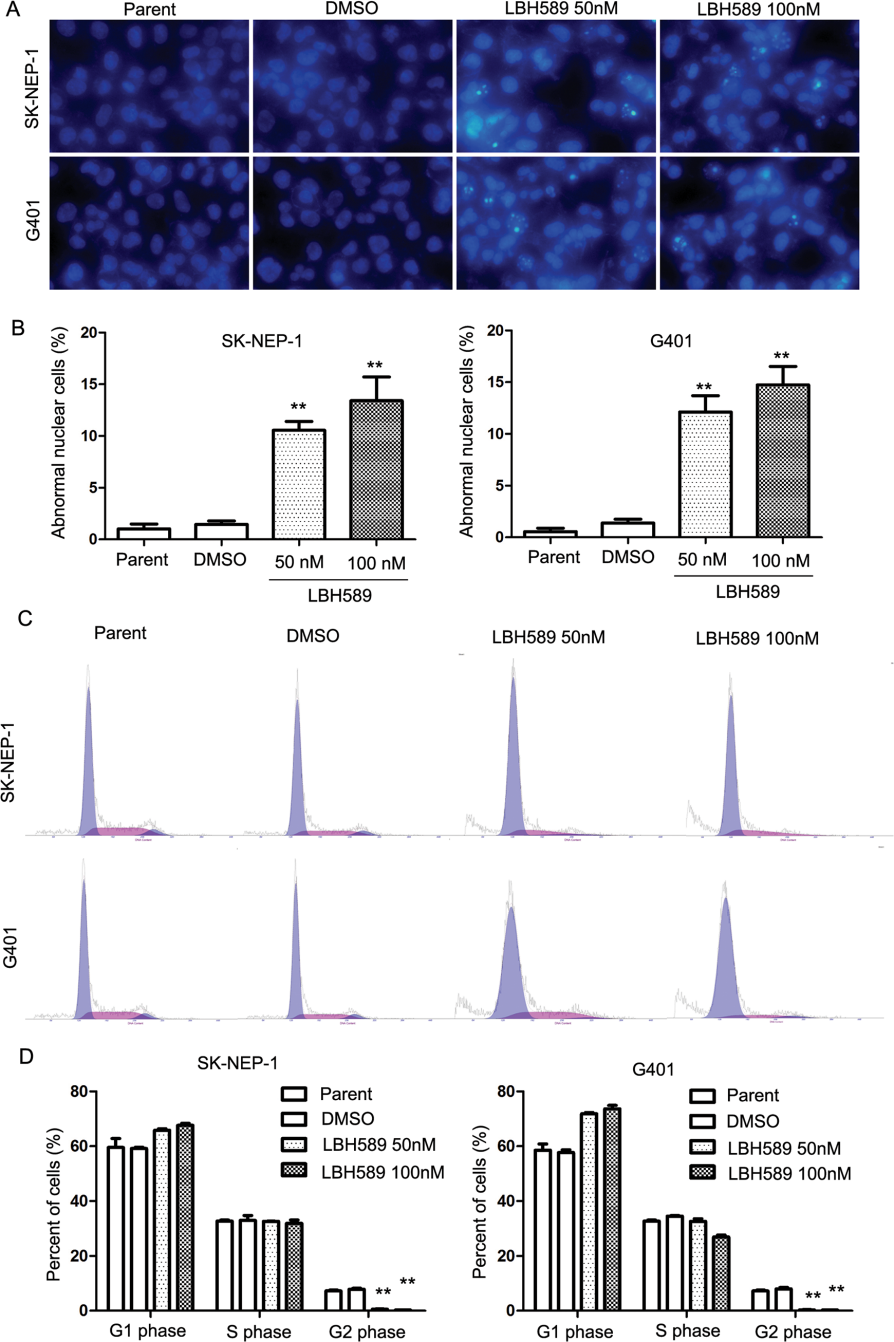
LBH589 induced DNA fragmentation and cell cycle disorder in SK-NEP-1 and G401 cells. (A) Micrographs following Hoechst 33342 staining of cells treated with LBH589 (50 nM and 100nM) for 24 h. This demonstrates micrograph shows the induction of DNA fragmentation and abnormal nuclear cell formation. The abnormal nuclear cells increased significantly with LBH589 treatment compared with mock treatment in both SK-NEP-1 and G401 cells. ** *P* < 0.01. (B) The number of cells with abnormal nuclei increased significantly compared with the control group in both SK-NEP-1 (100nM 13.4% ± 3.99% *vs*. DMSO 1.47% ± 0.57%, respectively; *P* = 0.033) and G401 cells (100nM 14.73% ± 3.09% vs. DMSO1.40 ± 0.61%, respectively; *P* = 0.015). (C) Cell cycle analysis of SK-NEP-1 and G401 cells treated for 24 h with LBH589 at 50nM or 100nM compared with DMSO control mock treatment. The number of cells in the G_2_ phase of the LBH589 treatment group decreased significantly. (D) G_2_ phase in SK-NEP-1 (100nM 0.2% ± 0.0% *vs*. DMSO 7.81% ± 0.59%, respectively; *P* = 0.035) and G401 cells (100nM 0.18% ± 0.0% vs. DMSO7.96 ± 0.80%, respectively; *P* = 0.046). All analyses were repeated three times. ** *P* < 0.01.

Hoechst 33342 staining analysis showed DNA fragmentation and cells with abnormal nuclei after 24 hours of LBH589 treatment (Fig 3A). The number of cells with abnormal nuclei increased significantly compared with control group in both SK-NEP-1 (100nM 13.4% ± 3.99% *vs*. DMSO 1.47% ± 0.57%, respectively; *P* = 0.033) and G401 cells (100nM 14.73% ± 3.09% vs. DMSO1.40 ± 0.61%, respectively; *P* = 0.015) (Fig 3B).Cell cycle assay (Fig 3C) showed that the number of cells in the G_2_ phase was significantly down regulated in the LBH589 treatment group at both 50nM and 100nM doses, G_2_ phase in SK-NEP-1 (100nM 0.2% ± 0.0% *vs*. DMSO 7.81% ± 0.59%, respectively; *P* = 0.035) and G401 cells (100nM 0.18% ± 0.0% vs. DMSO7.96 ± 0.80%, respectively; *P* = 0.046)(Fig 3D).

**Fig 4:**
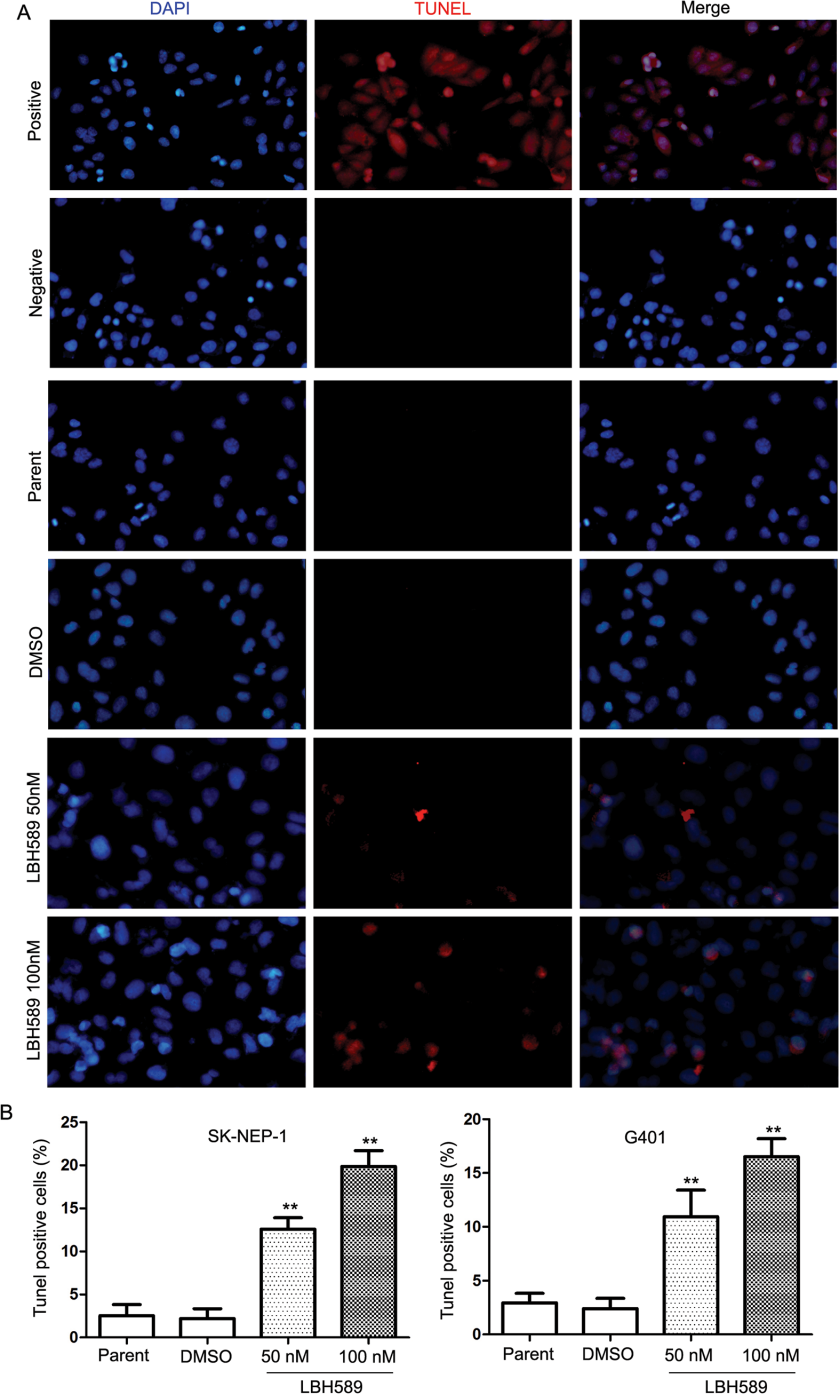
LBH589 induced DNA fragmentation in SK-NEP-1 and G401 cells. The TUNEL assay relies on the presence of nicks in the DNA that can be identified by terminal deoxynucleotidyl transferase (TdT), an enzyme that catalyzes the addition of dUTPs that are secondarily labeled with a marker. Micrographs showing TUNEL staining of cells treated with LBH589 (50 nM and 100 nM) for 24 h. Red cells demonstrates the induction of DNA fragmentation. (A) The DNA fragmentation increased significantly with LBH589 treatment compared with mock treatment in both SK-NEP-1 and G401 cells. (B) Results show the percentages of TUNEL positive cells in SK-NEP-1 (100nM 19.87% ± 3.19% vs. DMSO 2.20% ± 1.99%, respectively; *P* = 0.003); and G401 (100nM 16.53% ± 2.86% vs. DMSO2.40 ± 1.67%, respectively; *P* = 0.004) cells, ** *P* < 0.01.

TUNEL staining analysis showed more TUNEL positive cells in the LBH589 treatment groups (Fig 4A). Fig 4B shows the percentages of TUNEL positive cells in SK-NEP-1 (100nM 19.87% ± 3.19% vs. DMSO 2.20% ± 1.99%, respectively; *P* = 0.003); and G401 (100nM 16.53% ± 2.86% vs. DMSO2.40 ± 1.67%, respectively; *P* = 0.004) cells.

**Fig 5:**
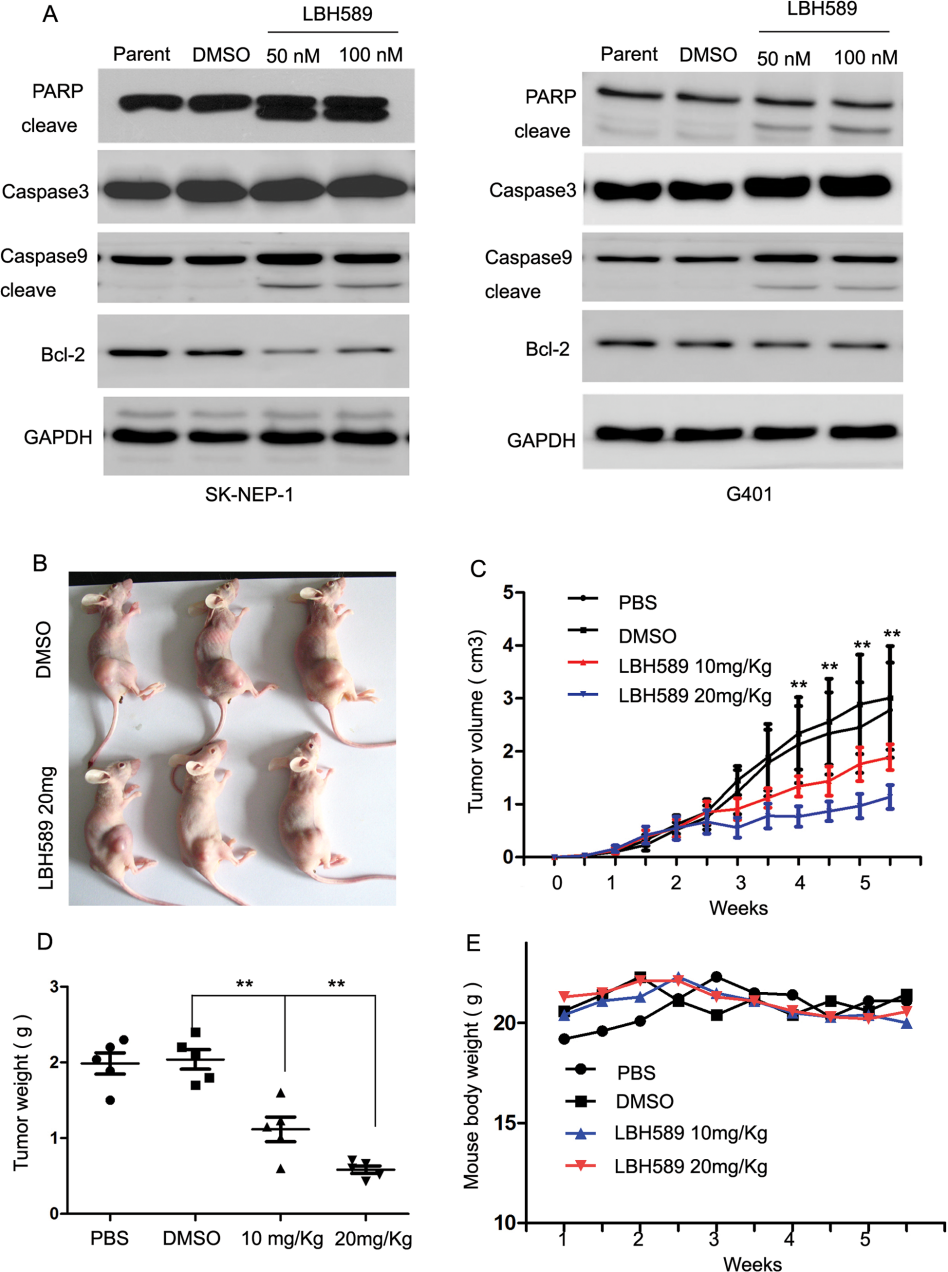
LBH589 treatment inhibited the growth of SK-NEP-1 xenograft tumors in nude mice. SK-NEP-1 cells were injected subcutaneously into five nude mice in every group. Ten days after injection, mice were treatment with PBS, DMSO, and LBH589 10mg/kg and 20mg/kg dose. (A) To clearly demonstrate that LBH589 causes apoptosis in SK-NEP-1 and G401 cells, we assessed the expression of PARP, caspase 3, caspase 9 and Bcl-2, recognized markers of apoptosis by western blotting. Down regulation of Bcl-2 and cleaved PARP and caspase 9 was observed after 24 hours of treatment with 50nM and 100nM LBH589. (B) SK-NEP-1 xenograft tumors from the treatment experiment. (C) Growth curve of SK-NEP-1 cells treated with LBH589, DMSO and PBS. LBH589 significantly inhibited the growth of SK-NEP-1 xenografts (LBH589 10mg/kg: 1.89 ± 0.77 cm^3^; LBH589 20mg/kg: 1.14 ± 0.55 cm^3^) compared with the DMSO group (DMSO: 3.01 ± 2.4 cm^3^) or PBS group (PBS: 2.78 ± 2.20 cm^3^, ANOVA P < 0.01). (D) Tumor weight of the treatment experiment. LBH589 treatment decreased the weight of the tumors (LBH589 10mg/kg: 1.12 ± 0.36 g; LBH589 20mg/kg: 0.59 ± 0.11 g) compared to DMSO group (DMSO: 2.04 ± 0.29 g) or PBS group (PBS: 1.98 ± 0.31 g, ANOVA P < 0.01). (E) Body mass curve analysis of nude mice in the treatment experiment. The body mass curve of nude mice treated with LBH589 was almost the same as the control group. At the end of the experiment, body weights of nude mice treated with LBH589 were almost same as the control group (LBH589 10mg/kg: 20.42 ± 2.43g; LBH589 20mg/kg: 20.56 ± 2.34g) compared with the DMSO group (DMSO: 21.44 ± 2.37g) or PBS group cells (PBS: 21.10 ± 1.39g, ANOVA P >0.05).

To clearly demonstrate that LBH589 causes apoptosis in SK-NEP-1 and G401 cells, we assessed the expression of PARP, caspase 3, caspase 9 and Bcl-2, recognized markers of apoptosis by western blotting. Down regulation of Bcl-2, cleaved PARP and caspase 9 was observed after 24 hours of treatment with 50nM and 100nM LBH589 (Fig 5A). This result is consistent with the data of Annexin V assay and the cell cycle analysis, demonstrating that LBH589 induced apoptosis in SK-NEP-1 and G401 cells. The results suggested that LBH589 has promising antitumor activity against SK-NEP-1 and G401 cells.

### LBH589 treatment inhibited the growth of SK-NEP-1 xenograft tumors in nude mice

The inhibition impact of LBH589 on the growth of SK-NEP-1 cells in nude mice was assessed. Each group, five nude mice were subcutaneously injected with SK-NEP-1 cells. Our results showed that LBH589 significantly inhibited the growth of SK-NEP-1 xenografts (LBH589 10mg/kg: 1.89 ± 0.77 cm^3^; LBH589 20mg/kg: 1.14 ± 0.55 cm^3^) compared with DMSO group (DMSO: 3.01 ± 2.4 cm^3^) or PBS group (PBS: 2.78 ± 2.20 cm^3^, ANOVA P < 0.01 Fig 5B and 5C). LBH589 treatment decreased the weight of the tumors (LBH589 10mg/kg: 1.12 ± 0.36 g; LBH589 20mg/kg: 0.59 ± 0.11 g) compared to DMSO group (DMSO: 2.04 ± 0.29 g) or PBS group (PBS: 1.98 ± 0.31 g, ANOVA P < 0.01 Fig 5D). We also observed that the body mass curve of nude mice treated with LBH589 were almost the same as the control group. At the end of experiment, body weight was LBH589 10mg/kg: 20.42 ± 2.43g; LBH589 20mg/kg: 20.56 ± 2.34g compared to the DMSO group (DMSO: 21.44 ± 2.37g) or PBS group (PBS: 21.10 ± 1.39g, ANOVA P >0.05 Fig 5E). These studies support the view that LBH589 has significant role and few side effects in the treatment of SK-NEP-1 xenograft tumors.

### Microarray analysis of differentially expressed genes in LBH589-treated SK-NEP-1 cells

The Arraystar_Human_LncRNA_8x60k v3.01 microarray was used to identify differentially expressed genes and lncRNAs in LBH589-treated SK-NEP-1 cells compared with DMSO-treated control cells. We have submitted our microarray analysis to the GEO repository and assigned GEO accession number is GSE64975.

**Fig 6:**
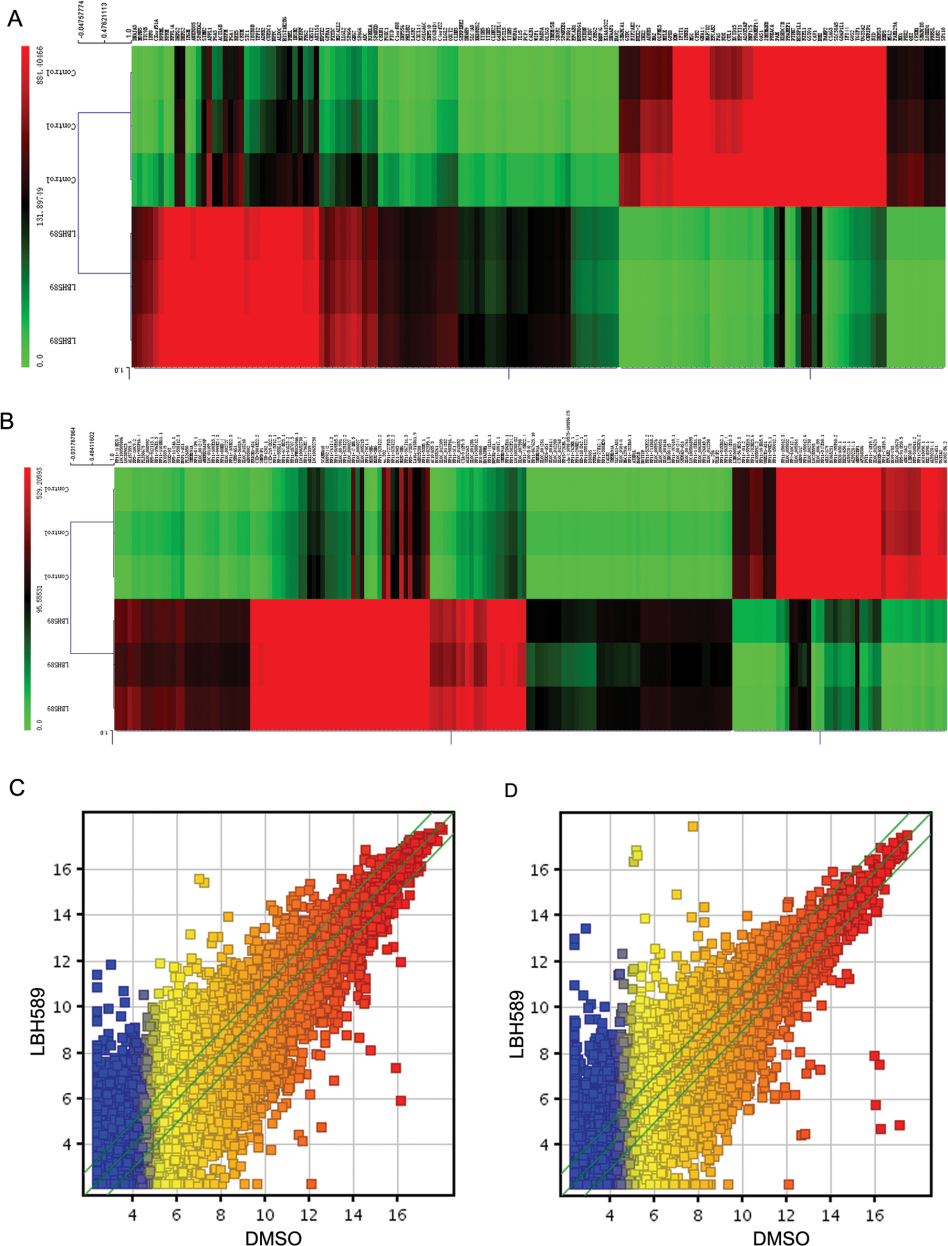
Cluster analysis of differentially expressed genes and lncRNAs in LBH589-treated SK-NEP-1 cells. The Arraystar_Human_LncRNA_8x60k v3.0 1 microarray was used to identify differentially expressed lncRNAs and mRNAs in LBH589-treated SK-NEP-1 cells compared to DMSO-treated control cells. (A) Hierarchical clustering analysis of the 664 significantly up regulated mRNAs and 632 significantly downregulated mRNAs ≥ 5- fold in LBH589-treated SK-NEP-1 cells. (B) Hierarchical clustering analysis of the differently expressed lncRNAs with a fold change ≥ 5-fold in LBH589-treated SK-NEP-1 cells. (C) Scatter-Plot assessing the mRNAs expression variation between DMSO-treated control cells and LBH589-treated SK-NEP-1 cells. The green lines are Fold Change Lines (The default fold change value given is 2.0). The mRNAs above the top green line and below the bottom green line indicated more than 2.0 fold change of mRNAs between the two compared samples. (D) Scatter-Plot assessing the lncRNAs expression variation between DMSO-treated control cells and LBH589-treated SK-NEP-1 cells. The green lines are Fold Change Lines (The default fold change value given is 2.0). The lncRNAs above the top green line and below the bottom green line indicated more than 2.0 fold change of lncRNAs between the two compared samples.

In the lncRNA/mRNA expression profiling data, we identified 6653 differently expressed mRNAs in LBH589-treated SK-NEP-1 cells (Fig 6A, S1 Dataset and S2 Dataset). Compared with DMSO-treated control cells, 664 mRNAs were significantly up regulated and 632 mRNAs were significantly down regulated > 5 fold changes in LBH589-treated SK-NEP-1 cells. Clustering analysis was used to visualize the relationships between the mRNA expression patterns present in the samples (fold changes ≥ 5; Fig 6B). In the lncRNA expression profiling data, we analyzed a total of 33327 lncRNAs expressed in SK-NEP-1 cells, of which 8135 were differentially expressed in LBH589-treated SK-NEP-1 cells (Fig 6C, S3 Dataset and S4 Dataset). Hierarchical clustering analysis of the differently expressed lncRNAs with a fold change ≥ 5-fold is presented in Fig 6D.

### Gene ontology and KEGG Pathway analysis of differentially expressed mRNAs in LBH589-treated SK-NEP-1 cells

**Fig 7:**
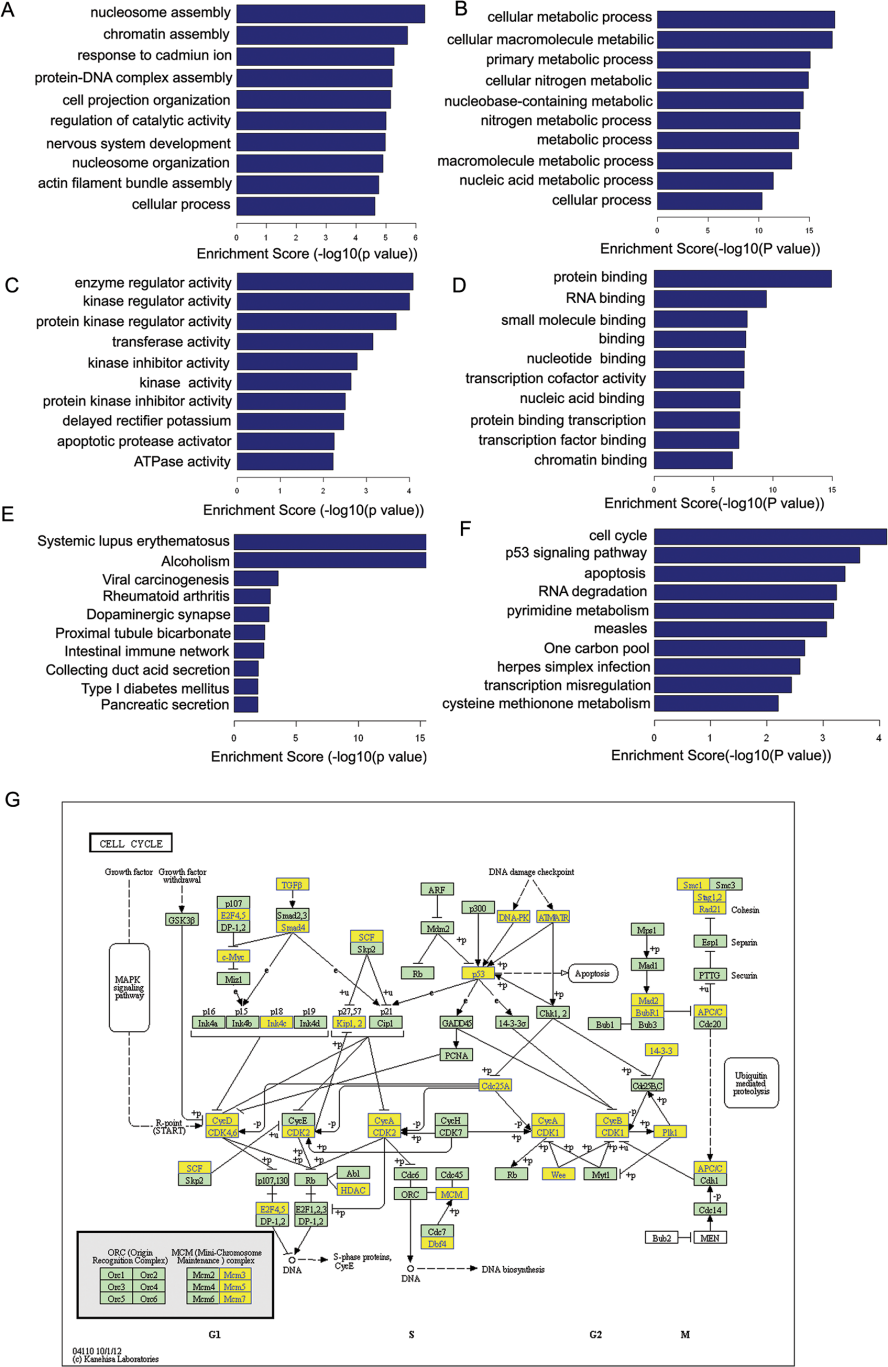
Gene ontology and KEGG Pathway analysis of differentially expressed mRNAs in LBH589-treated SK-NEP-1 cells. (A) The most enriched GO terms targeted by the up regulated transcripts were involved in a variety of functions. (B) The most enriched GO terms targeted by the down regulated transcripts were involved in a variety of functions. (C) The most significant Cellular component (CC) Enrichment scores for up regulated transcripts. (D) The most significant Cellular component (CC) Enrichment scores for down regulated transcripts. (E) The most significant Molecular function (MF) Enrichment scores for up regulated transcripts. (F) The most significant Molecular function (MF) Enrichment scores for down regulated transcripts. (G) KEGG pathway annotations of the most enriched pathways, cell cycle, with a p-value of 7.48007E^-05^. The cell cycle pathway proteins included *CCNA2*, *CCNB2*, *CCND1*, *CCND2*, *CDK4*, *CDKN1B*, *HDAC2*, etc.

We performed ontological pathway enrichment analysis for the differently expressed genes and gene product enrichment with particular attention to GO biological processes and molecular function. Biological processes enrichment analysis was performed using the DAVID tool to gain insights into their functions. The most enriched GOs targeted by the up regulated and down regulated transcripts were involved in a variety of functions including nucleosome assembly, chromatin assembly, cellular metabolic process and cellular macromolecule metabolic (Fig 7A and 7B). The most significant Cellular component (CC) Enrichment scores are shown in Fig 7C and 7D, and the most significant Molecular function (MF) Enrichment scores are shown in Fig 7E and 7F.

The KEGG database was used to investigate the pathways in which the differentially expressed genes are involved. KEGG annotations of the most enriched pathways are shown in Fig 7G, with a p value of 7.48007E-05. Specifically, the significant pathways included cell cycle, p53 signaling pathway and apoptosis. The cell cycle pathway included proteins such as *CCNA2*, *CCNB2*, *CCND1*, *CCND2*, *CDK4*, *CDKN1B* and *HDAC2* (S5 Dataset).

### Ingenuity pathway analysis of differentially expressed mRNAs in LBH589-treated SK-NEP-1 cells

**Fig 8:**
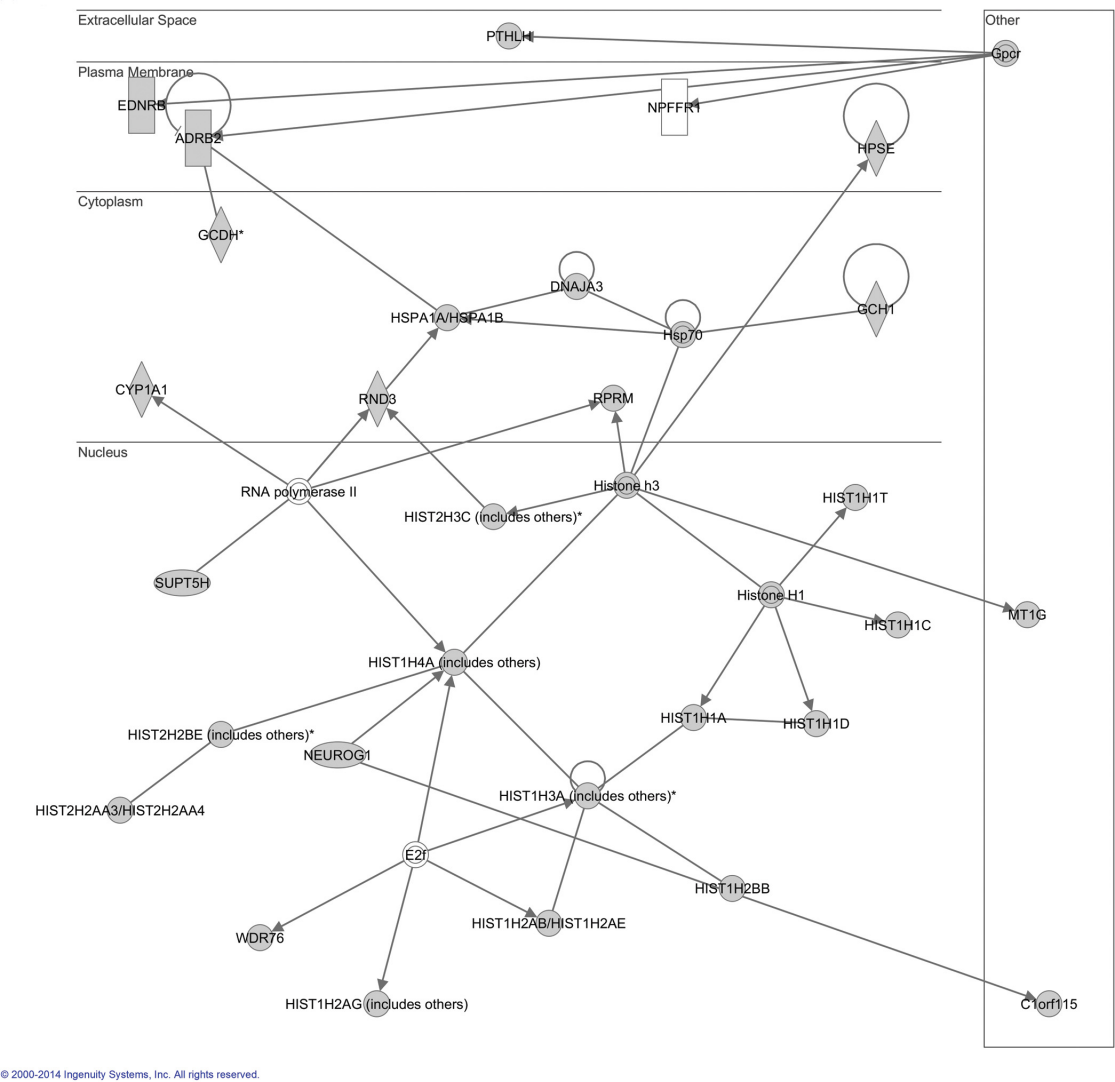
Ingenuity Pathways Analysis (IPA) summary of up regulated genes in SK-NEP-1 cells treated with LBH589. To investigate possible interactions of differently regulated genes, datasets representing up regulated genes in SK-NEP-1 cells treated with LBH589 were imported into the Ingenuity Pathway Analysis Tool. The following data are shown: most highly rated network in the IPA analysis and the network representation of the most highly rated network. Statistical analysis determined that the shaded genes are significant. A solid line represents a direct interaction between the two gene products and a dotted line means there is an indirect interaction.

**Fig 9:**
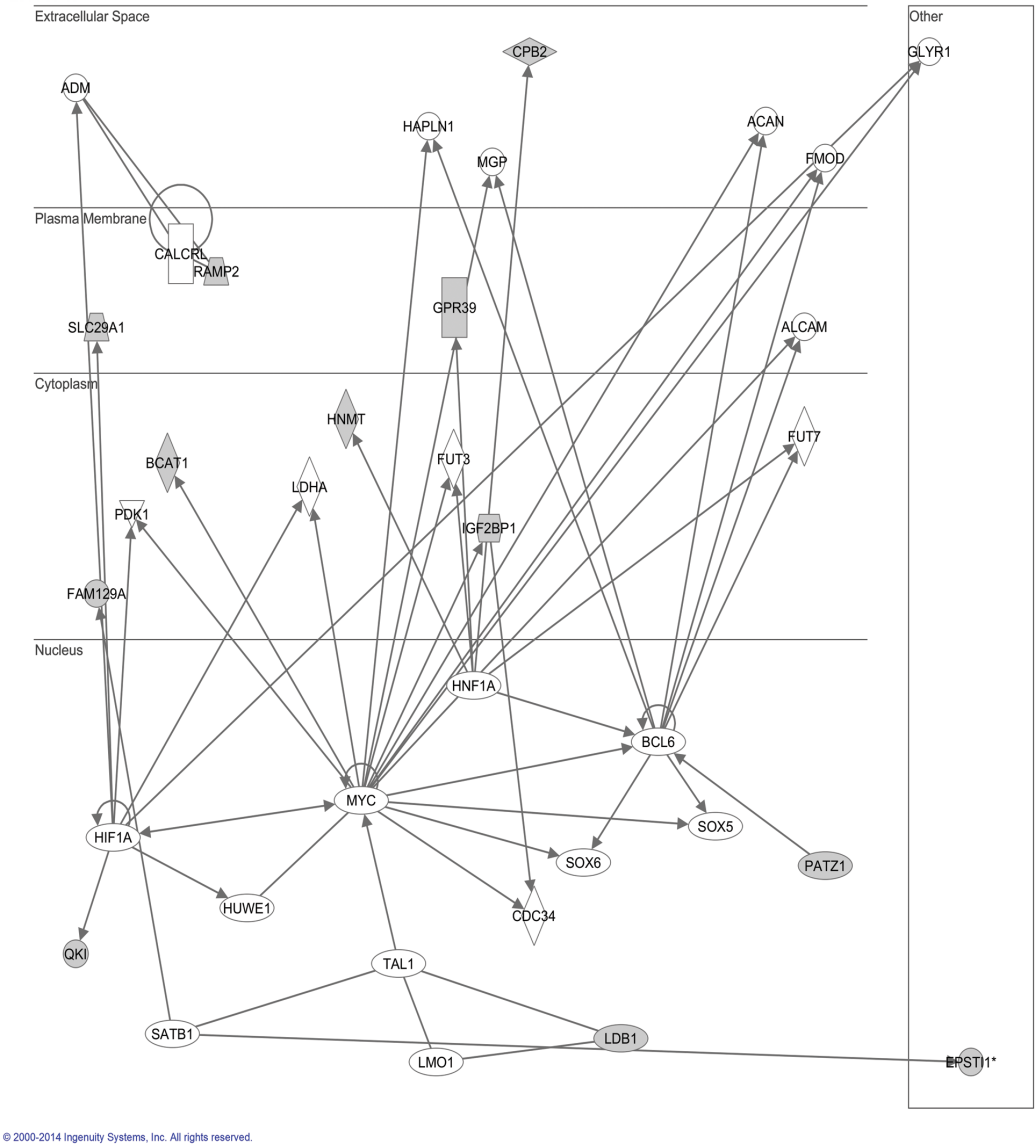
Ingenuity Pathways Analysis (IPA) summary of down regulated genes in SK-NEP-1 cells treated with LBH589. To investigate possible interactions of differently regulated genes, datasets representing down regulated genes in SK-NEP-1 cells treated with LBH589 were imported into the Ingenuity Pathway Analysis Tool. The following data are shown: most highly rated network in IPA analysis. The network representation of the most highly rated network. Statistical analysis determined that the shaded genes are significant. A solid line represents a direct interaction between the two gene products and a dotted line means there is an indirect interaction.

To investigate the possible biological interactions of the proteins encoded by the differentially regulated genes, datasets representing genes with altered expression profiles derived from microarray analyses were imported into the Ingenuity Pathway Analysis Tool. Ingenuity Pathway Knowledge Base (IPKB) is derived from known functions and interactions of genes or proteins published. The list of differentially expressed genes analyzed by IPA revealed significant networks. Fig 8 represents the highest rated network using the up regulated genes in LBH589-treated SK-NEP-1 cells. Fig 9 represents the highest rated network using down regulated genes in LBH589-treated SK-NEP-1 cells. IPA analysis showed that the important upstream molecules included *HIST2H3C*, *HIST1H4A*, *HIST1A*, *HIST1C*, *HIST1D*, Histone H1, Histone H3, *RPRM*, *HSP70* and *MYC*. These upstream regulators such as Histone H1, Histone H3 and the Histone family have already been reported as important regulators for LBH589 treatment. Histones have been widely investigated and are important targets of LBH589. However, there has been no report about the relationship between *RPRM*, *HSP70*, *MYC* and LBH589. This work is the first time to indicate that *RPRM*, *HSP70* and *MYC* may be important regulators during LBH589 treatment. Thus, these results provide new clues to the molecular mechanism of apoptosis induced by LBH589.

### Real-time PCR and western blot analysis validation of the apoptosis-regulated genes in LBH589-treated SK-NEP-1 cells

**Fig 10:**
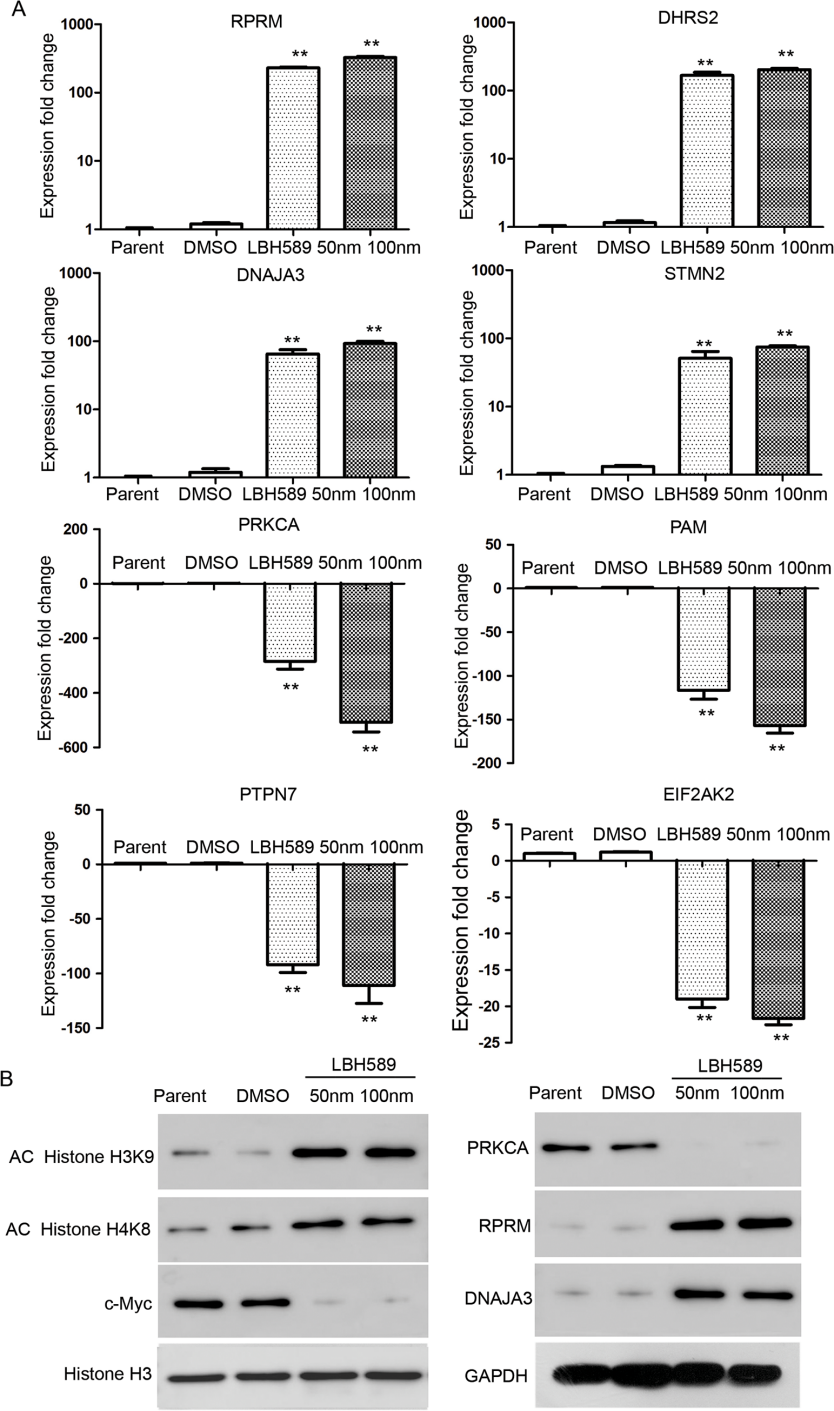
Real-time PCR and western blot analysis validation of LBH589-regulated genes in LBH589-treated SK-NEP-1 cells. (A) Quantitative RT-PCR analysis of *RPRM*, *DHRS2*, *DNAJA3*, *STMN2*, *PRKACA*, *PAM*, *PTPN7* and *EIF2AK2* in LBH589-treated SK-NEP-1 cells. (B) Western blot analysis of AC Histone H3K9, AC Histone H4K8, Histone H3, c-MYC, PRKCA, RPRM and DNAJA3 in LBH589-treated SK-NEP-1 cells.

To validate the expressions of apoptosis-regulated genes in LBH589-treated SK-NEP-1 cells, we analyzed the expression of eight apoptosis-regulated genes in LBH589-treated SK-NEP-1 cells: *RPRM*, *DHRS2*, *DNAJA3*, *STMN2*, *PRKACA*, *PAM*, *PTPN7* and *EIF2AK2*. Fig 10A showed that the qRT-PCR analysis results were consistent with the microarray analysis. Western blot analysis showed that LBH589 induces hyperacetylation of histone H3K9 and H4K8, and down regulates the expression of c-MYC. Western blot analysis also validated the apoptosis-regulated genes PRKCA, RPRM and DNAJA3 in LBH589-treated SK-NEP-1 cells (Fig 10B).

Up to 90% of wilms tumor patients can be cured with current therapy, but there is still need to improve therapy for aggressive patients [35]. Various HDACs have bee reported involved in different functions and pathways in cells. HDACs expression levels vary in different kinds of cancer cells. HDAC1 has been reported over expressed in gastric and prostate cancers. In lung, colon, esophageal and breast cancers high expression of HDAC1 also indicates poor prognosis. High expression level of HDAC2 was also reported in cervical, colorectal and gastric cancers [36–38]. HDAC3 has also been reported over expressed in prostate, gastric and colorectal cancer patients [39–41]. Over expression of HDAC8 in neuroblastoma correlates with metastasis and poor prognosis. Expression level of HDAC11 is also up regulated in rhabdomyosarcoma [42–44]. The present study indicated that HDACs are also important targets for human wilms tumor cells.

Several structurally diverse HDAC inhibitors have been developed as cancer therapeutic agents and have been shown to cause differentiation, cell cycle arrest, or apoptosis *in vitro* [15]. Now, there are over 490 clinical trials for cancer to investigate the clinical application of HDAC inhibitors. Vorinostat (suberoylanilide hydroxamic acid, SAHA), the first HDAC inhibitor for refractory and relapsed cutaneous T-cell lymphoma (CTCL) has been approved by FDA [45, 46]. LBH589 is a novel pan HDACs inhibitor that possesses potent growth inhibitory activity in Ph(-) ALL cells [47], triple-negative breast cancer (TNBC)[24], hepatocellular carcinoma [29], prostate cancer (PCa) cells [48], relapsed/refractory Waldenstrom macroglobulinemia (WM) [49] and multiple myeloma (MM) [50]. However, to date, the molecular function of LBH589 in WT has remained unknown.

Our results indicated that LBH589 treatment caused inhibition of cell proliferation of SK-NEP-1 and G401 cells in a dose-dependent manner. Cells treated with LBH589 showed more apoptotic feature and abnormal nuclei compared with the control group. In the LBH589 treatment group the G_2_ phase was significantly down regulated, there were more TUNEL positive cells and cleaved PARP, caspase 9 were observed. These results demonstrated for the first time that LBH589 induced apoptosis in SK-NEP-1 and G401 cells. Our research also indicated that LBH589 treatment inhibits the growth of SK-NEP-1 xenograft tumors in nude mice.

These results supported the view that LBH589 has a significant role and few side effects in the treatment of SK-NEP-1 xenograft tumors.

In this study, we analyzed the apoptosis-regulated genes by LBH589 using the Arraystar Human LncRNA Array. LncRNA/mRNA expression profiling data identified 6653 differentially expressed mRNAs in LBH589-treated SK-NEP-1 cells. 8135 lncRNAs were differentially expressed in LBH589-treated SK-NEP-1 cells. This is the first report of lncRNA/mRNA expression profiling related to LBH589 treatment in SK-NEP-1 cells. Our research will focus on the molecule function of these LBH589-related lncRNAs.

Ontological pathway enrichment analysis showed the most enriched GOs targeted by the up regulated and down regulated transcripts were involved in a variety of functions including nucleosome assembly, chromatin assembly, cellular metabolic process and cellular macromolecule metabolic. KEGG pathway annotations of the most enriched pathways identified the cell cycle-related proteins, such as *CCNA2*, *CCNB2*, *CCND1*, *CCND2*, *CDK4*, *CDKN1B* and *HDAC2*. Ingenuity Pathway Analysis identified important upstream molecules such as *HIST2H3C*, *HIST1H4A*, *HIST1A*, *HIST1C*, *HIST1D*, Histone H1, Histone H3, *RPRM*, *HSP70* and *MYC*. Previously, Jove et al showed that LBH589 induces hyperacetylation of histone H3K9 and H4K8 in Ph^-^ acute lymphoblastic leukemia cells [47]. Our research also supported the hyperacetylation function of LBH589 in WT cells. However, our results also indicated that *RPRM*, *HSP70* and *MYC* might be important regulators during LBH589 treatment. Reprimo (*RPRM*), initially identified as a downstream effecter of p53-induced cell cycle arrest at G_2_/M, is a putative tumor suppressor gene that is silenced *via* promoter methylation in several types of human cancer [51–54]. In 83 primary human gastric cancer tissues, *RPRM* gene promoter methylation was cancer-specific and frequently observed. Enforced *RPRM* expression robustly inhibited cell proliferation and anchorage-independent colony formation, as well as enhanced DNA damage-induced apoptosis [55]. *RPRM* may be new target of LBH589, and our result also implied a new network for LBH589 for the first time.

## Conclusions

The present study demonstrated that LBH589 treatment resulted in apoptosis and inhibition of cell proliferation of SK-NEP-1and G401 cells. LBH589 had a significant role and few side effects in the treatment of SK-NEP-1 xenograft tumors. Arraystar Human LncRNA Array analysis provided the expression profile of lncRNAs/mRNAs in LBH589-treated SK-NEP-1 cells. GO, KEGG and IPA analysis identified new targets and network affected by LBH589 treatment. *RPRM*, *HSP70* and *MYC* may be important regulators during the LBH589 treatment. The results may provide new clues to the molecular mechanism of apoptosis induced by LBH589.

## Supporting Information

S1 Dataset Differentially Expressed mRNAs upregulated in SK-NEP-1 cells treated with LBH589. (XLS)

S2 Dataset Differentially Expressed mRNAs downregulated in SK-NEP-1 cells treated with LBH589. (XLS)

S3 Dataset Differentially Expressed LncRNAs upregulated in SK-NEP-1 cells treated with LBH589. (XLS)

S4 Dataset Differentially expressed LncRNAs downregulated in SK-NEP-1 cells treated with LBH589. (XLS)

S5 Dataset Summary of KEGG analysis the differentially expressed mRNAs in SK-NEP-1 cells treated with LBH589. (XLS)
